# Evaluation of potassium-enriched biochar and GA3 effectiveness for Improving wheat growth under drought stress

**DOI:** 10.1186/s12870-023-04613-0

**Published:** 2023-12-05

**Authors:** Ghulam Sarwar, Tauseef Anwar, Mehvish Malik, Haseeb ur Rehman, Subhan Danish, Tahani Awad Alahmadi, Mohammad Javed Ansari

**Affiliations:** 1 Department of Botany, The Islamia University of Bahawalpur, Bahawalpur, Pakistan; 2 Department of Agronomy, Faculty of Agricultural Sciences and Technology, Bahauddin Zakariya University, Multan, Punjab, Pakistan; 3 Department of Soil Science, Faculty of Agricultural Sciences and Technology, Bahauddin Zakariya University, Multan, Punjab, Pakistan; 4 Department of Pediatrics, College of Medicine and King Khalid University Hospital, Medical City, King Saud University, PO Box-2925, 11461 Riyadh, Saudi Arabia; 5 Department of Botany, Hindu College Moradabad, (MJP Rohilkhand University Bareilly), Moradabad, India 244001; 6 Al-Waili Foundation of Science, New York, USA

**Keywords:** Activated carbon, Chlorophyll contents, Gibberellic acid, Growth attributes, Osmotic stress, Wheat

## Abstract

Osmotic stress is a significant concern in agricultural crop production as it can harm crop growth, development, and productivity. Agriculture crops are particularly vulnerable to osmotic stress due to their reliance on water availability for various physiological processes. Organic amendments like activated carbon biochar and growth hormone gibberellic acid (GA3) can play a vital role. However, the time needed is to modify the established amendment to achieve better results. That’s why the current study used potassium-enriched biochar (KBC = 0.75%) with and without GA3 (15 mg/L) as amendments under no osmotic stress and osmotic stress in wheat. Results showed that GA3 + KBC caused significant enhancement in germination (9.44%), shoot length (29.30%), root length (21.85%), shoot fresh weight (13.56%), shoot dry weight (68.38), root fresh weight (32.68%) and root dry weight (28.79%) of wheat over control under osmotic stress (OS). A significant enhancement in chlorophyll a, chlorophyll b and total chlorophyll, while the decline in electrolyte leakage of wheat, also validated the effectiveness of GA3 + KBC over control in OS. In conclusion, GA3 + KBC is the most effective among all applied treatments for improving wheat growth attributes under no osmotic and osmotic stress. Further research is needed at the field level, focusing on various cereal crops, to establish GA3 + KBC as the optimal treatment for effectively mitigating the impacts of osmotic stress.

**Supplementary Information:**

The online version contains supplementary material available at 10.1186/s12870-023-04613-0.

## Introduction

Osmotic stress under changing climatic conditions poses a significant threat to crop production [1] by imposing an imbalance in the osmotic potential between plant cells and the surrounding environment, leading to water deficit or excess [2–4]. This stress condition adversely affects crops by impeding water uptake and restricting the availability of essential nutrients [5–8]. Consequently, plants experience wilting, stunted growth and reduced productivity [9, 10]. Moreover, osmotic stress induces cellular damage due to water deficiency, causing cell dehydration, membrane impairment, and disruption of vital cellular processes [11, 12]. This cellular damage further contributes to diminished photosynthesis, metabolic dysfunction, and decreased crop yield [13, 14]. Additionally, osmotic stress weakens plants, rendering them more susceptible to pest infestations and diseases, exacerbating the yield reduction [13, 15]. Disruptions in metabolic and biochemical processes, including enzyme synthesis, protein activity, and hormonal regulation, further impede plant growth and development [16, 17]. To mitigate these adverse impacts of osmotic stress, activated carbon biochar usage as an amendment is becoming popular [18, 19].

Biochar is a carbon-rich material produced by the pyrolysis of organic biomass, such as crop residues or wood waste [20]. When activated, biochar undergoes a process that enhances its porosity, surface area, and adsorption capacity [9]. These properties make activated carbon biochar effective in improving soil water-holding capacity and mitigating the impacts of osmotic stress on crops [9]. By improving water availability to plant roots, activated carbon biochar helps alleviate the water deficit associated with osmotic stress, enabling crops to withstand dry conditions and maintain optimal growth and productivity [21]. Studies have also shown that exogenous application of gibberellic acid (GA3) can enhance the tolerance of crops to osmotic stress [5]. GA3 treatment has been shown to alleviate the negative effects of water deficit on seed germination and seedling establishment [22]. It promotes faster and more uniform germination by breaking seed dormancy and stimulating cell elongation, enabling seedlings to emerge from the soil under adverse conditions [18]. GA3 also improves root growth and development, enhancing the capacity of plants to explore the soil for water uptake, even in water-limited environments [23].

Moreover, GA3 has been reported to regulate stomatal behavior, which influences plant water status and transpiration rate [21]. Under osmotic stress conditions, plants tend to close their stomata to minimize water loss, which can also restrict carbon dioxide uptake for photosynthesis [24]. In addition to the above, Potassium maintains the osmotic balance within plant cells [25]. It plays a crucial role in osmoregulation by regulating the movement of water across cell membranes [22]. Potassium ions (K +) accumulate in the cytoplasm, increasing the osmotic potential and facilitating water uptake by plant cells [26]. This helps to counteract the water deficit caused by osmotic stress and maintain turgor pressure within the cells, thereby preventing wilting and sustaining proper plant growth [24].

Wheat, an integral part of human diets for millennia, is a nutritional powerhouse revered for its versatility and affordability [27]. This grain offers a rich tapestry of nutrients, including complex carbohydrates for sustained energy, dietary fiber for digestive health, and a decent plant-based protein source [1, 27]. Packed with essential B vitamins and minerals, wheat fuels various bodily functions while being a cornerstone in diverse culinary traditions worldwide, from bread and pasta to cultural staples. However, osmotic stress played an imperative role in decreasing its growth and productivity [7].

Previous research has extensively examined the individual effects of potassium biochar and GA3 on plant responses to osmotic stress. However, there is a lack of comprehensive studies investigating their combined effects [28]. Therefore, this study provides novel insights into the potential synergistic or additive effects of KBC and GA3 on wheat growth and stress tolerance, shedding light on the development of effective strategies for enhancing crop performance in osmotically challenging environments [26]. The combined use of GA3 and KBC is hypothesized to effectively mitigate wheat’s osmotic stress by regulating chlorophyll contents and antioxidants [29].

That’s why the current study was conducted with aims to evaluate the impact of potassium biochar (KBC) with and without GA3 on growth attributes, chlorophyll contents, and antioxidants of wheat cultivated under no osmotic stress and osmotic stress. The current study aims to fill the knowledge gap by evaluating the impact of potassium biochar (KBC) with and without GA3 on growth attributes, chlorophyll contents, and antioxidants of wheat cultivated under both non-osmotic stress and osmotic stress conditions.

## Material and methods

### Experimental site and design

The study was conducted in the research area of Botany Department, Islamia Univeristy Bahawalpur, Bahawalpur Punjab, Pakistan to assess the effects of different treatments on wheat plants under varying drought levels. The treatments included different combinations of potassium-enriched biochar (KBC) and gibberellic acid (GA3) applied at different drought stress levels. The experiment followed a completely randomized design (CRD) with four replications (4 pots per treatment). A total of 32 pots were used, each containing 8 kg of soil. The pre-experimental characteristics of soil are provided in Table 1.

**Table 1:** Pre-experimental soil, biochar, and irrigation characteristics

Soil	Values	Biochar	Values	Irrigation	Values
pH	8.27	pH	7.07	pH	7.21
EC*e* (dS/m)	3.01	EC*e* (dS/m)	5.11	EC (µS/cm)	211
SOC (%)	0.60	Volatile Matter (%)	20	Carbonates (meq./L)	0.00
TN (%)	0.03	Fixed carbon (%)	65	Bicarbonates (meq./L)	5.56
EP (mg/kg)	5.81	Ash content (%)	15	Chloride (meq./L)	0.10
AK (mg/kg)	166	TN (%)	0.09	Ca + Mg (meq./L)	3.79
Sand (%)	25	TP (%)	0.41	Sodium (mg/L)	163
Silt (%)	40	Particle Size	< 2 mm	TN = Total Nitrogen EP = Extractable Phosphorus AK = Available Potassium Ca = Calcium; Mg = Magnesium	
Clay (%)	35	EC = Electrical Conductivity TN = Total Nitrogen TP = Total Phosphorus			
Texture	Clay Loam				

### Potassium enriched biochar (KBC) preparation and application

First, fruit waste was collected from the local fruit market as the carbon source for biochar production. The biomass material was then dried and mixed with potassium at a 2% level based on the weight of the biomass using potassium sulphate salt (Product Number: 223492, Batch Number: WXBD9938V, Brand: SIGALD, CAS Number: 7778–80-5,). Next, the potassium-enriched biomass material was subjected to pyrolysis at 550 °C, which involved heating the material in the absence of oxygen for 75 min. After the pyrolysis process, the KBC was cooled and stored in suitable containers. It is essential to store biochar in a dry and well-ventilated area to maintain its properties and prevent moisture absorption. A KBC amendment was added at a rate of 0.75% (w/w) basis to the respective treatment posts. The KBC was thoroughly mixed with the soil to ensure proper incorporation. The characteristics of KBC are provided in Table 1.

### Seeds collection and sterilization

The seeds of wheat variety Dilkash 2020 were purchased from the certified seed dealer of Bahawalpur. The seeds were subjected to a sterilization by soaking them in a sterilizing solution of 5% sodium hypochlorite. This solution effectively disinfects the seeds and minimizes the risk of fungal or bacterial contamination. After sterilization, thorough rinsing with sterile distilled water was performed 4 times. This ensured that the seeds were not only pathogen-free but also free from any chemical residues of 5% sodium hypochlorite [30].

### Pots dimension and seeds sowing

For experimental purpose clay pots were used having dimension 8 inch wide and 12 inch depth. In each pot 8 kg of soil was added. A total of 10 seeds were sown in each pot. After germination 4 healthy seedlings were maintained in each pot [25].

### Treatments plan

The experiment consisted of eight treatments, including control and different combinations of GA3 and KBC under normal and drought stress conditions. The treatments were as follows: Control (No drought/osmotic stress), 15 ppm GA3 + 0.75% KBC (No drought/osmotic stress), 15 ppm GA3 (No drought/osmotic stress), 0.75% KBC (No drought/osmotic stress), Drought stress (35% Field Capacity), 15 ppm GA3 + 0.75% KBC (35% Field Capacity), 15 ppm GA3 (35% Field Capacity) and 0.75% KBC (35% Field Capacity). All treatments were applied in 4 replicates following completely randomized design (CRD).

### Fertilizer

In every pot macro-nutrient needs, nitrogen (N), phosphorus (P), and potassium (K) fertilizers were incorporated at the recommended rate of 120:90:60 kg ha^−1^ [31]. Urea was added in three splits i.e., sowing, seedling (21 days old) and stem elongation (40 days old). In the case of diammonium phosphate (DAP) and muriate of potash (MOP) fertilizers, the recommended application rates were employed in a single dose during the sowing process.

### Osmotic stress

Drought stress was induced by maintaining the soil moisture level at 35% of field capacity (w/w), simulating water-limited conditions. The control treatments were maintained at optimal soil moisture levels (65% field capacity) [25].

### Harvesting and data collection

Soon after harvesting (60 days old plants when vegetative growth was completed and tillering was just to began) various growth parameters i.e., shoot length, root length, shoot fresh and dry weight, root fresh and dry weight. The dry weight was determined by subjecting the plant material to a drying process in an oven maintained at a temperature of 65 °C for a duration of 48 h. An analytical grade balance was employed for the measurement of both root and shoot fresh and dry weights.

### Chlorophyll contents, electrolyte leakage and antioxidants

In the spectrophotometric analysis using the Arnon method, a 80% acetone solution was employed as the solvent for chlorophyll extraction from the plant samples. The final volume of the extraction solution was adjusted to 10 ml [32]. Final values were conputed using the folloqing eq.

$$\begin{array}{l}\text{Chlorophyll a} \left(\frac{\mathrm{mg}}{\mathrm{g}}\right)=\frac{\left(12.7 \times \mathrm{ A}663\right)-\left(2.69 \times \mathrm{ A}645\right)\times \mathrm{V}}{1000 \times \mathrm{W}}\\ \text{Chlorophyll b} \left(\frac{\mathrm{mg}}{\mathrm{g}}\right)=\frac{\left(22.9 \times \mathrm{ A}645\right)-\left(4.68 \times \mathrm{ A}645\right)\times \mathrm{V}}{1000 \times \mathrm{W}}\\ \text{Total Chlorophyll }\left(\frac{\mathrm{mg}}{\mathrm{g}}\right)= 20.2\left(\mathrm{OD }645\right)+8.02\left(\mathrm{OD }663\right)\times \mathrm{V}/1000 (\mathrm{W})\end{array}$$

The electrolyte leakage was determined using standardized EC meter [33].

$$\text{Electrolyte Leakage }\left(\mathrm{\%}\right)= (\mathrm{C}2 -\mathrm{ C}1) /\mathrm{ C}1\mathrm{ x }100$$

Where initial electrical conductivity (C1); final electrical conductivity (C2) after sample exposure water bath at 121 °C for 20 min.

Antioxidant activity was assessed by measuring the activity of peroxidase (POD) [34], superoxide dismutase (SOD) [35], and catalase (CAT) [36] enzymes. Data were recorded for each parameter from all replicate pots.

### Statistical analysis

The collected data were subjected to standard statistical analysis [37]. Mean comparison was performed using appropriate statistical tests (Fisher’s LSD), and significance was considered at *p* < 0.05 using OriginPro 2021 [38]. Paired comparison, and cluster plots were also made by using OriginPro 2021.

## Results

### Germination percentage

**Fig. 1:**
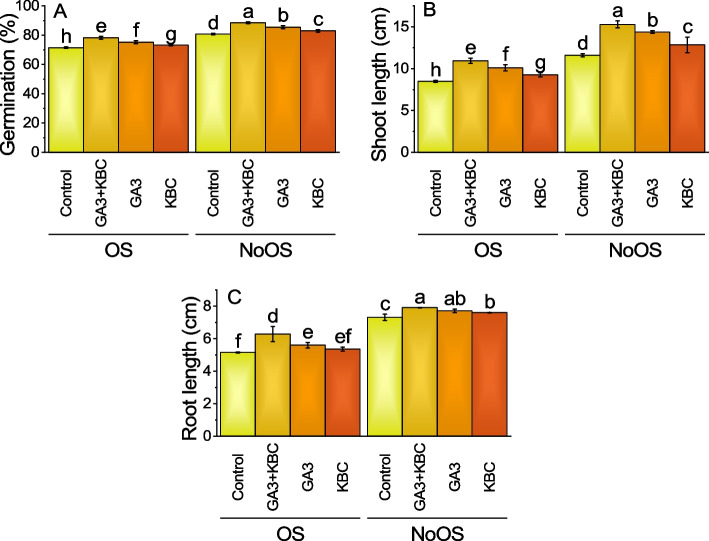
Effect of gibberellic acid (GA3) and Potassium enriched biochar (KBC) sole and combined application on germination (**A**), shoot length (**B**) and root length (**C**) of wheat cultivated under osmotic stress. Bars ± SE (Fisher’s LSD; *p* ≤ 0.05) are means of 4 replicates (4 pots per treatment). Different letters on bars showed significant alteration

Main effect of GA3 and KBC was significant while interactive effect of GA3 × KBC was non-significant for the germination of seeds (Table S1). The GA3 + KBC (gibberellic acid and potassium-enriched biochar) treatment exhibited the highest increase, with a percentage change of 9.44% for the germination of seeds. This indicates a significant improvement in germination compared to the control group. The GA3 treatment resulted in a 5.24% increase, while the KBC treatment showed a 2.45% increase in germination. Under the NoOS (no stress) condition, all treatments also demonstrated increases in germination compared to the control group. The GA3 + KBC treatment had the highest percentage change at 9.60%, indicating a substantial improvement in germination. The GA3 treatment resulted in a 5.88% increase, and the KBC treatment showed a 2.79% increase in germination (Fig. 1A; Table S15).

### Shoot length

Main and intercatiove effects of GA3 and KBC was significant for shoot length (Table S2). When GA3 + KBC treatment was added, the mean shoot length increased to 10.96 cm, representing a 29.30% increase compared to the control. Similarly, the GA3 treatment resulted in a mean shoot length of 10.10 cm, indicating a significant 19.27% increase. The KBC treatment showed a slightly lower mean shoot length of 9.26 cm, with a 9.27% increase compared to the control group. Turning to the NoOS treatments, the control group had a mean shoot length of 11.60 cm, reflecting no specific treatment applied. However, when GA3 + KBC treatment was administered, the mean shoot length increased to 15.29 cm, signifying a substantial 31.74% increase compared to the control group. Similarly, the GA3 treatment showed a mean shoot length of 14.39 cm, indicating a significant 24.02% increase. The KBC treatment yielded a slightly lower mean shoot length of 12.85 cm, resulting in a 10.75% increase compared to the control group (Fig. 1B; Table S16).

### Root length

Main and intercatiove effects of GA3 and KBC was significant for root length (Table S3). In the OS treatments, the control group showed an average root length of 5.16 cm, and the application of GA3 + KBC treatment increased by 21.85%, compared to the control. GA3 alone produced a mean root length of 5.60 cm, demonstrating an 8.58% increase, whereas, KBC resulted in a mean root length of 5.36 cm, indicating a 3.92% rise. For the NoOS treatments, the control group had an average root length of 7.32 cm, and the administration of GA3 + KBC treatment caused an 8.03% increase. GA3 alone resulted in a mean root length of 7.72 cm, demonstrating a 5.46% rise; KBC yielded a mean root length of 7.61 cm, signifying a 3.93% increase compared to the control group (Fig. 1C; Table S17).

### Shoot fresh weight

**Fig. 2:**
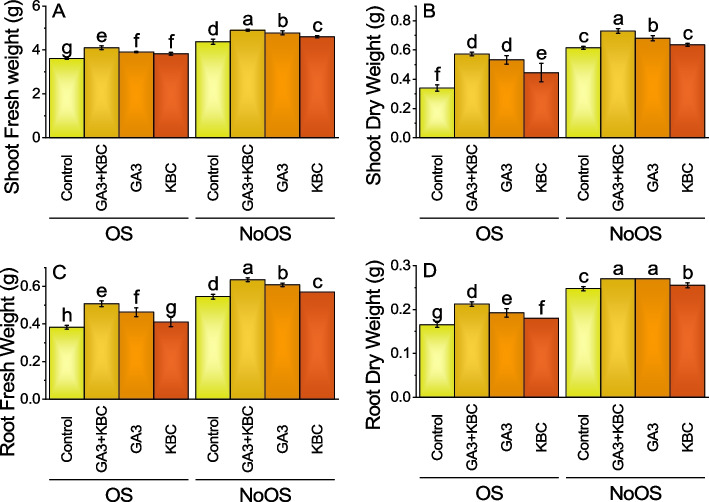
Effect of gibberellic acid (GA3) and Potassium enriched biochar (KBC) sole and combined application on shoot fresh weight (**A**), shoot dry weight (**B**) root fresh weight (**C**) and root dry weight (**D**) of wheat cultivated under osmotic stress. Bars ± SE (Fisher’s LSD; *p* ≤ 0.05) are means of 4 replicates (4 pots per treatment). Different letters on bars showed significant alteration

Main effect of GA3 and KBC was significant while interactive effect of GA3 × KBC was non-significant for shoot fresh weight (Table S4). Under the OS, the control group exhibited a mean shoot fresh weight of 3.61 g. The application of GA3 + KBC treatment led to a significant increase, with the mean shoot fresh weight reaching 4.10 g, corresponding to a percentage change of 13.56% compared to the control. The GA3 treatment also increased, with a mean shoot fresh weight of 3.91 g, showing a percentage change of 8.30%. Similarly, the KBC treatment showed an improvement, with a mean shoot fresh weight of 3.83 g, indicating an increase of 6.09%. Under the NoOS condition, the control group had a higher mean shoot fresh weight of 4.37 g. The GA3 + KBC treatment further increased the shoot fresh weight to 4.91 g, reflecting a percentage change of 12.11%. Similarly, the GA3 treatment resulted in a mean shoot fresh weight of 4.78 g, showing an improvement of 9.26%. The KBC treatment exhibited a mean shoot fresh weight of 4.61 g, representing a percentage change of 5.26% (Fig. 2A; Table S18).

### Shoot dry weight

Main and intercatiove effects of GA3 and KBC was significant for shoot dry weight (Table S5). In OS, control treatment had a mean of 0.34 g while GA3 + KBC treatment had a mean of 0.57 g which is a 68.38% increase from control under OS for shoot dry weight. Treatment GA3 in OS had a mean of 0.53 g which is a 56.62% increase from control. Under OS, KBC treatment had a mean of 0.45 g, a 30.88% increase from the OS Control treatment for shoot dry weight. In case of NoOS, control treatment had a mean of 0.61 g; in NoOS, GA3 + KBC treatment had a mean of 0.73, an 18.70% increase from control. At NoOS, GA3 treatment had a mean of 0.68 g, a 10.57% increase in shoot dry weight from the NoOS control treatment. The NoOS KBC treatment had a mean of 0.64 g, a 3.25% increase from the NoOS Control treatment. Overall, all the treatments increased the shoot dry weight from the Control treatment, with the highest increase being 68.38% (Fig. 2B; Table S19).

### Root fresh weight

Main effect of GA3 and KBC was significant while interactive effect of GA3 × KBC was non-significant for root fresh weight (Table S6). The application of GA3 + KBC treatment resulted in a significant increase, with a root fresh weight of 0.51 g, corresponding to a percentage change of 32.68% compared to the control under OS. The GA3 treatment also exhibited a notable increase, with a root fresh weight of 0.46 g, representing a percentage change of 20.92%. Similarly, the KBC treatment showed improvement, with a root fresh weight of 0.41 g, indicating a percentage change of 7.19%. Under NoOS conditions, the control group had a higher root fresh weight of 0.54 g. The application of GA3 + KBC treatment further increased the root fresh weight to 0.63 g, reflecting a percentage change of 16.51%. Similarly, the GA3 treatment resulted in a root fresh weight of 0.61 g, showing an 11.47% increase. The KBC treatment exhibited a root fresh weight of 0.57 g, representing an increase of 4.59% (Fig. 2C; Table S20).

### Root dry weight

Main and intercatiove effects of GA3 and KBC was significant for root dry weight (Table S7). The application of GA3 + KBC treatment led to a significant increase, with a root dry weight of 0.21 g, corresponding to a percentage change of 28.79% compared to the control in OS. Similarly, the GA3 treatment resulted in a root dry weight of 0.19 g, showing an increase of 16.67%. The KBC treatment also demonstrated an effect, with a root dry weight of 0.18 g, indicating a percentage increase of 9.09%. In NoOS conditions, the control group had a root dry weight of 0.25 g. The application of GA3 + KBC treatment slightly increased the root dry weight by 9.09%. Similarly, the GA3 and KBC treatments resulted in a root dry weight of 0.27 g and 0.26 g, respectively, indicating a percentage change of 9.09% and 3.03% (Fig. 2D; Table S21).

### Chlorophyll a

**Fig. 3:**
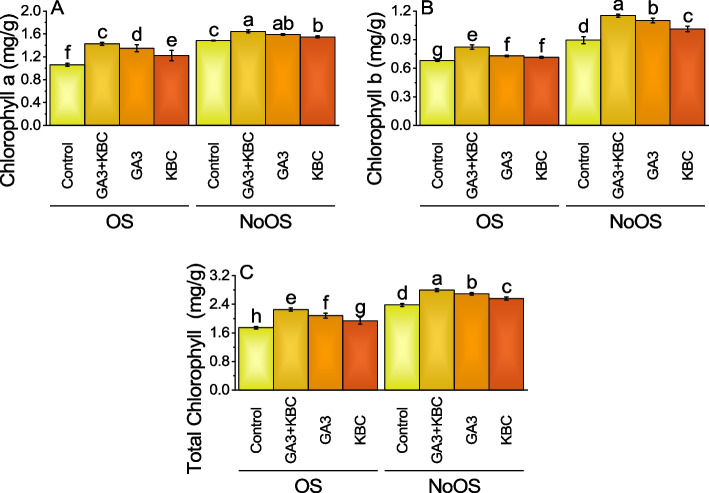
Effect of gibberellic acid (GA3) and Potassium enriched biochar (KBC) sole and combined application on chlorophyll a (**A**), chlorophyll b (**B**) and total chlorophyll (**C**) of wheat cultivated under osmotic stress. Bars ± SE (Fisher’s LSD; *p* ≤ 0.05) are means of 4 replicates (4 pots per treatment). Different letters on bars showed significant alteration

Main and intercatiove effects of GA3 and KBC was significant for chlorophyll a (Table S8). For the control under OS, the chlorophyll a weight was 1.0625. The application of GA3 + KBC treatment caused a substantial increase to 1.4275, with a 34.35% change, while GA3 and KBC treatments respectively raised it to 1.35 and 1.2225, with a 27.06% and 15.06% change. Under NoOS, the control had a higher chlorophyll a weight of 1.485. GA3 + KBC treatment further increased it to 1.6425, which was a 10.61% change, and GA3 and KBC treatments respectively raised it to 1.59 and 1.5475, representing 7.07% and 4.21% changes (Fig. 3A; Table S22).

### Chlorophyll b

Main and intercatiove effects of GA3 and KBC was significant for chlorophyll b (Table S9). During OS, the control group displayed a mean chlorophyll b value of 0.68. Application of the GA3 + KBC treatment led to a higher mean chlorophyll b value of 0.8225, indicating a percentage change of 20.96% compared to the control. Similarly, the GA3 treatment resulted in a mean chlorophyll b value of 0.73, signifying a change of 7.35%. The KBC treatment also had an impact, with a mean chlorophyll b value of 0.715 and a change of 5.15%. In case of NoOS, the control group exhibited a relatively higher mean chlorophyll b value of 0.895. The application of the GA3 + KBC treatment further elevated the mean chlorophyll b value to 1.1525, reflecting a change of 28.77%. Likewise, the GA3 treatment yielded a mean chlorophyll b value of 1.1, indicating a change of 22.91%. The KBC treatment resulted in a mean chlorophyll b value of 1.0125, representing a change of 13.13% (Fig. 3B; Table S23).

### Total chlorophyll

Main effect of GA3 and KBC was significant while interactive effect of GA3 × KBC was non-significant for total chlorophyll (Table S10). Under the OS condition, the GA3 + KBC treatment exhibited a 29.12% increase in total chlorophyll compared to the control group, indicating a significant improvement. The GA3 treatment resulted in a 19.37% increase, while the KBC treatment showed an 11.19% increase in total chlorophyll. When considering the NoOS, the GA3 + KBC treatment demonstrated a 17.44% increase in total chlorophyll compared to the control group, indicating a notable improvement. The GA3 treatment resulted in a 13.03% increase, and the KBC treatment showed a 7.56% increase in total chlorophyll (Fig. 3C; Table S24).

### Electrolyte leakage

**Fig. 4:**
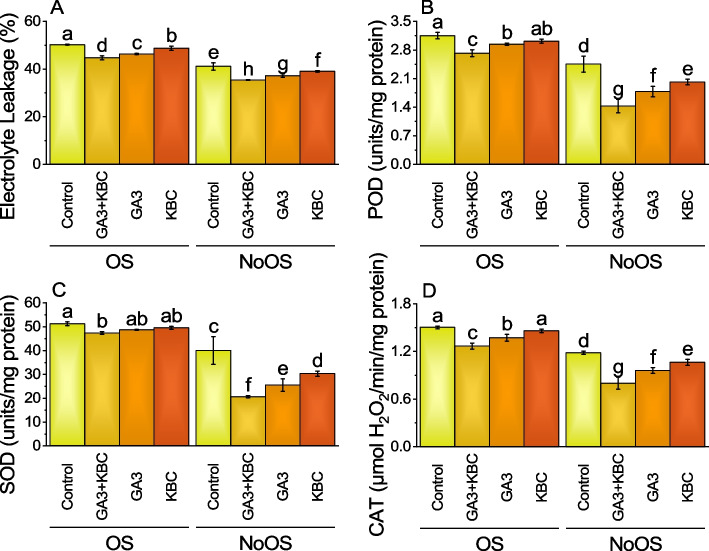
Effect of gibberellic acid (GA3) and Potassium enriched biochar (KBC) sole and combined application on electrolyte leakage (**A**), POD (**B**) SOD (**C**) and CAT (**D**) of wheat cultivated under osmotic stress. Bars ± SE (Fisher’s LSD; *p* ≤ 0.05) are means of 4 replicates (4 pots per treatment). Different letters on bars showed significant alteration

Main effect of GA3 and KBC was significant while interactive effect of GA3 × KBC was non-significant for electrolyte leakage (Table S11). The initial values of the control group for the OS and NoOS conditions were 50.26% and 41.13%, respectively. The GA3 + KBC treatment displayed a -11.02% decrease in EL under the OS condition. The GA3 treatment led to a -7.74% decrease, and the KBC treatment indicated a -3.02% reduction. When evaluating the NoOS, the GA3 + KBC treatment revealed a -13.89% reduction in EL compared to the control group. The GA3 treatment yielded a -9.48% decrease, and the KBC treatment demonstrated a -5.10% decrease in electrolytic leakage (Fig. 4A; Table S25).

### Peroxidase (POD)

Main and intercatiove effects of GA3 and KBC was significant for POD (Table S12). In case of OS, the GA3 + KBC treatment exhibited a -13.72% decrease in POD activity compared to the control group, indicating a significant reduction. The GA3 treatment resulted in a -6.66% decrease, while the KBC treatment showed a -4.28% decrease in POD activity. When considering the NoOS, the GA3 + KBC treatment demonstrated a -41.85% decrease in POD activity compared to the control group, indicating a substantial reduction. The GA3 treatment resulted in a -27.39% decrease, and the KBC treatment showed a -17.92% decrease in POD activity. Please note that the initial values of the control group for the OS and NoOS conditions were 3.1525 and 2.455, respectively (Fig. 4B; Table S26).

### Superoxide Dismutase (SOD)

Main and intercatiove effects of GA3 and KBC was significant for SOD (Table S13). The GA3 + KBC treatment showed a -7.56% drop in SOD activity under the OS compared to the control group 51.2625, showing a small reduction. Superoxide dismutase activity was reduced by -5.00% with the GA3 treatment and by -3.29% with the KBC treatment. Compared with the control group 40.0225, the GA3 + KBC treatment showed a -48.47% drop in SOD activity in the NoOS condition, indicating a substantial reduction. Superoxide dismutase activity was reduced by -36.19% with the GA3 therapy and by -24.32% with the KBC treatment (Fig. 4C’ Table S27).

### Catalase (CAT)

Main and intercatiove effects of GA3 and KBC was significant for CAT (Table S14). When the GA3 + KBC treatment was applied, the CAT value decreased by 15.64% under OS. Similarly, the application of GA3 alone resulted in 8.65% decrease in CAT, while the KBC treatment led to a 2.83% decrease in CAT compared to the control group in OS. Applying GA3 + KBC treatment caused a significant decrease of 32.35% to a CAT over control in NoOS. Similarly, the CAT value for GA3 treatment representing a 18.82% decrease, while the KBC treatment resulted in 10.15% decrease compared to the control group under NoOS (Fig. 4D; Table S28).

### Cluster plot convex hull

**Fig. 5:**
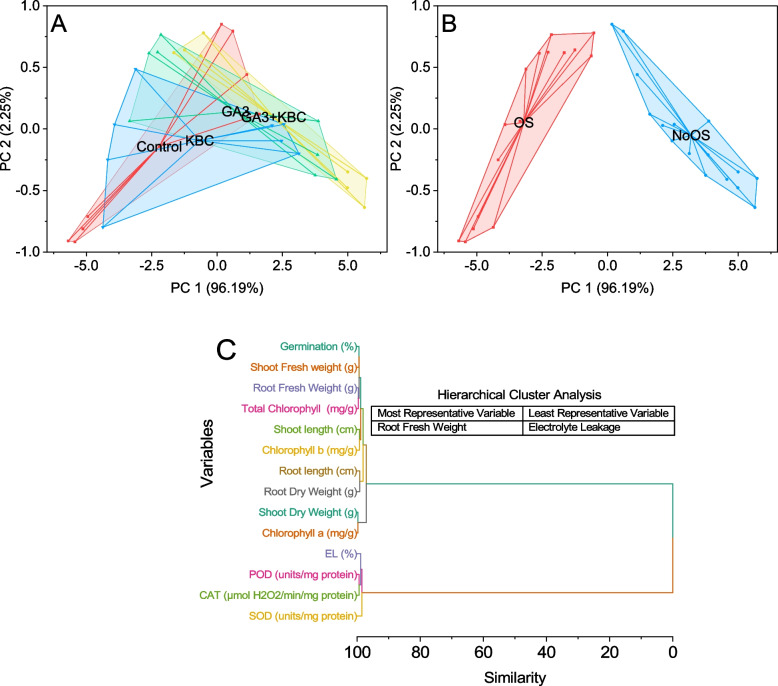
Cluster plot convex hull for treatments (**A**), osmotic stress (**B**) and hierarchical cluster plot (**C**) for studied attributes

One cluster, labeled as Control, is enclosed within a convex hull and consists of data points with PC1 scores ranging from approximately -5.7 to 1.6 and PC2 scores ranging from -0.92 to 0.85. Another cluster, GA3 + KBC, encompasses a broader range of data points with PC1 scores ranging from approximately -5.7 to 5.7 and PC2 scores ranging from -0.92 to 0.85. This GA3 + KBC cluster further exhibits two subclusters, GA3 + KBC and GA3, based on variations in their PC1 scores. The GA3 subcluster is confined within its convex hull. It comprises data points with PC1 scores ranging from approximately -2.6 to 4.6 and PC2 scores ranging from -0.38 to 0.77. Meanwhile, the GA3 + KBC subcluster shares the same convex hull as the main GA3 + KBC cluster, including data points from both the GA3 + KBC and GA3 treatments. The KBC cluster is also encapsulated within its convex hull, encompassing data points with PC1 scores ranging from approximately -4.4 to -2.4 and PC2 scores ranging from -0.8 to 0.49. Similar to the GA3 + KBC cluster, the KBC cluster exhibits two subclusters. The first KBC subcluster has a convex hull encompassing data points with PC1 scores ranging from approximately -4.4 to -3.9 and PC2 scores ranging from -0.8 to 0.04. The second KBC subcluster is enclosed within its convex hull. It includes data points with PC1 scores ranging from approximately -3.9 to 2.6 and PC2 scores ranging from -0.25 to 0.85 (Fig. 5A). There are two clusters identified in the plot. The first cluster, labeled as OS (osmotic stress), is enclosed within a convex hull and consists of data points with PC1 scores ranging from approximately -5.7 to -2.1 and PC2 scores ranging from -0.92 to 0.78. The second cluster, labeled as NoOS (no osmotic stress), is also enclosed within a convex hull and includes data points with PC1 scores ranging from approximately 0.17 to 5.72 and PC2 scores ranging from -0.64 to 0.85 (Fig. 5B).

### Herarchical cluster plot

Shoot Dry Weight (g) and Chlorophyll a (mg/g) have the highest similarity value of 0.29739, suggesting a strong relationship between these two variables. Similarly, Root Fresh Weight (g) and Total Chlorophyll (mg/g) have a similarity value of 0.37816, indicating their close association. The variables Germination (%) and Shoot Fresh Weight (g) share a similarity value of 0.56431, indicating a moderate level of similarity. Another notable similarity is observed between Shoot length (cm) and Chlorophyll b (mg/g) with a similarity value of 0.75097, indicating a strong relationship. The variables POD (units/mg protein) and CAT (µmol H2O2/min/mg protein) have a similarity value of 0.63187, suggesting a significant association between these two variables. The strong relationships observed between shoot dry weight and chlorophyll a, as well as between shoot length and chlorophyll b, emphasize the integral role of chlorophyll in plant growth and development. Similarly, the close association between root fresh weight and total chlorophyll content suggests that these parameters respond cohesively to environmental factors. The moderate similarity between germination percentage and shoot fresh weight indicates a nuanced connection that warrants further exploration. Furthermore, the significant association between peroxidase (POD) and catalase (CAT) activities highlights the cooperative enzymatic responses of these factors (Fig. 5C).

### Pearson correlation

**Fig. 6:**
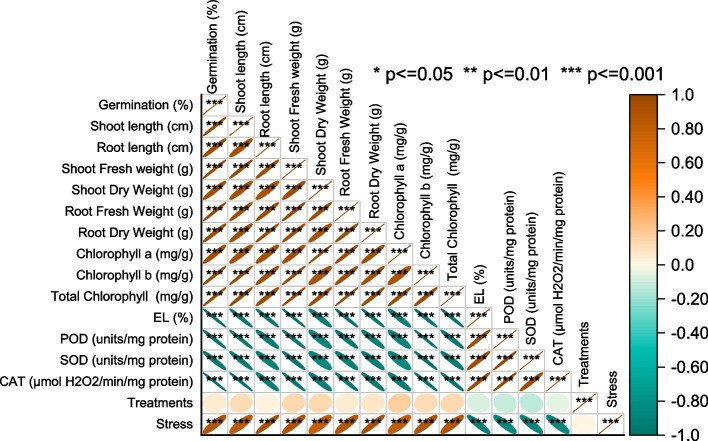
Pearson correlation for the studied attributes

Among the variables, Germination (%) shows a strong positive correlation with Stress (0.87114), indicating that higher levels of stress are associated with increased germination percentages. Similarly, Germination (%) also exhibits a positive correlation with Shoot length (cm) (0.83769), Root length (cm) (0.93775), Shoot Fresh weight (g) (0.89716), and several other variables, suggesting that these factors tend to co-vary positively with germination percentages. Furthermore, variables such as Chlorophyll a (mg/g) and Chlorophyll b (mg/g) show positive correlations with multiple variables, including Shoot length (cm), Shoot Fresh weight (g), and Total Chlorophyll (mg/g). These correlations suggest a potential relationship between chlorophyll content and plant growth-related variables. On the other hand, variables like EL (%) (electrolyte leakage), POD (units/mg protein) (peroxidase activity), SOD (units/mg protein) (superoxide dismutase activity), and CAT (µmol H2O2/min/mg protein) (catalase activity) exhibit negative correlations with various variables. This indicates an inverse relationship, suggesting that as the values of these variables increase, the measured variables tend to decrease (Fig. 6).

## Discussion

### Osmotic stress impact on plants

Osmotic stress triggers the production of reactive oxygen species (ROS) in plant cells, resulting in oxidative damage [39]. The use of KBC and GA3 together stimulates the enzymes that fight with free radicals, such as peroxidase (POD), superoxide dismutase (SOD), and catalase (CAT) [40]. These enzymes act as ROS scavengers, preventing cellular damage and maintaining cellular homeostasis [41]. As a consequence of this, electrolyte leakage, which functions as an indication of membrane integrity, is decreased, suggesting that the membrane's capacity to tolerate stress has increased. In addition to this, GA3 has been linked to improved antioxidant defense systems in plants [42]. Osmotic stress accumulates reactive oxygen species (ROS), causing oxidative damage to plant cells [43]. GA3 treatment has been found to increase the activity of antioxidant enzymes such as superoxide dismutase (SOD), catalase (CAT), and peroxidase (POD) [44]. These enzymes help scavenge ROS and protect plant cells from oxidative stress, reducing cellular damage and improving stress tolerance [45].

### Role of GA3

One of the key roles of GA3 is its involvement in seed germination and seedling establishment under osmotic stress conditions [23]. Osmotic stress often prevents seeds from germinating and delays the emergence of seedlings. On the other hand, research has shown that GA3 is effective in promoting germination that is both quicker and more uniform, therefore overcoming seed dormancy and increasing early seedling development [46]. This phenomenon facilitates the more effective establishment of plants in osmotic stress. In addition, gibberellic acid (GA3) plays a vital role in the regulation of stomatal behavior, a critical aspect of maintaining plant water balance. Plants have a tendency to shut their stomata in conditions of osmotic stress in order to mitigate water loss. Nevertheless, the application of GA3 has been shown to enhance the process of stomatal opening, so easing the exchange of gases and ultimately leading to an improvement in the water-use efficiency of plants [47]. The modulation of stomatal conductance enables plants to effectively manage the trade-off between water saving and carbon dioxide intake for photosynthesis, even when subjected to osmotic stress conditions [29].

### Role of K-enriched Biochar (KBC)

The combined application of potassium-enriched biochar (KBC) and GA3 has shown the potential to mitigate the adverse effects of osmotic stress on plants by regulating various physiological parameters [16]. One such parameter is chlorophyll content, which is crucial for photosynthetic efficiency. Osmotic stress often leads to a decline in chlorophyll a, chlorophyll b, and total chlorophyll levels [48]. Another mechanism through which KBC mitigates osmotic stress is by enhancing water retention in the soil [18]. Its porous structure acts as a reservoir for water, preventing rapid soil desiccation during dry spells. This increased water availability is crucial for maintaining adequate hydration levels in plant cells. KBC indirectly reduces electrolyte leakage by promoting soil moisture retention and providing a stable environment for plant roots [3]. This, in turn, helps maintain the integrity of cell membranes, reducing the loss of electrolytes from plant cells. Potassium (K) is an essential macronutrient for plants, playing a vital role in maintaining ionic balance and osmotic potential within plant cells [21]. KBC releases potassium ions slowly into the soil, ensuring a steady supply of this nutrient to plants. This helps in counteracting the adverse effects of osmotic stress by regulating osmotic pressure. On the other hand, POD is an enzyme that plays a pivotal role in scavenging reactive oxygen species (ROS) produced under osmotic stress conditions. KBC indirectly enhances the activity of POD by improving soil structure and nutrient availability. This enzyme helps in detoxifying harmful ROS, reducing oxidative damage to plant cells [49].

### Combined role of GA3 and KBC

The combined application of GA3 and KBC enhances the interaction between plant roots and the soil matrix. GA3 promotes root growth, while KBC provides a supportive environment with improved nutrient availability. This synergy leads to stronger, healthier root systems [50]. Applying KBC and GA3 has increased the fresh and dry weight of roots and shoots in stressed plants [50]. Additionally, shoot and root length can be positively influenced by KBC and GA3, facilitating better nutrient and water uptake. The improved growth parameters can improve overall plant performance and stress adaptation. These findings highlight the potential of KBC and GA3 as effective strategies for enhancing crop performance and productivity in water-limited environments [16]. Applying KBC and GA3 can help maintain higher chlorophyll contents, ensuring optimal photosynthetic activity even under stress conditions. Additionally, carotenoids, which play a role in photoprotection and antioxidant defense, can be positively influenced by KBC and GA3, further contributing to stress tolerance [42]. Potassium (K) significantly enhances plant growth and development under osmotic stress conditions [44].

## Conclusion

It is concluded that GA3 + KBC, is most effective among all applied treatments for improving wheat growth attributes under no osmotic and osmotic stress. Adding GA3 and KBC as the sole application can also improve chlorophyll contents. Yet, the performance of GA3 + KBC was significantly better over control. Regulation of POD, SOD, and CAT due to adding GA3 + KBC validated its effectiveness against osmotic stress. A significant decline in electrolyte leakage due to GA3 + KBC was the most representative variable that brought positive changes in wheat under osmotic stress. Growers should incorporate GA3 + KBC treatment to achieve better wheat growth under osmotic stress. More investigations are suggested at the field level on different cereal crops for the declaration of GA3 + KBC as best treatment for the mitigation of osmotic stress.

### Supplementary Information

**Additional file 1:** **Table S1.** Overall ANOVA for Gemination (%). **Table S2.** Overall ANOVA for Shoot length (cm). **Table S3.** Overall ANOVA for Root length (cm). **Table S4.** Overall ANOVA for Shoot fresh weight (g). **Table S5.** Overall ANOVA for Shoot dry weight (g). **Table S6.** Overall ANOVA for Root fresh weight (g). **Table S7.** Overall ANOVA for Root dry weight (g). **Table S8.** Overall ANOVA for Chlorophyll a (mg/g). **Table S9.** Overall ANOVA for Chlorophyll b (mg/g). **Table S10.** Overall ANOVA for Total chlorophyll (mg/g). **Table S11.** Overall ANOVA for Electrolyte leakage (%).**Table S12.** Overall ANOVA for POD (units/mg protein). **Table S13.** Overall ANOVA for SOD (units/mg protein). **Table S14.** Overall ANOVA for CAT (units/mg protein). **Table S15.** Fisher Test Raw Data for Germination. **Table S16.** Fisher Test Raw Data for Shoot length. **Table S17.** Fisher Test Raw Data for Root length. **Table S18.** Fisher Test Raw Data for Shoot fresh weight. **Table S19.** Fisher Test Raw Data for Shoot dry weight. **Table S20.** Fisher Test Raw Data for Root fresh weight. **Table S21.** Fisher Test Raw Data for Root dry weight. **Table S22.** Fisher Test Raw Data for Chlorophyll a. **Table S23.** Fisher Test Raw Data for Chlorophyll b. **Table S24.** Fisher Test Raw Data for Total chlorophyll. **Table S25.** Fisher Test Raw Data for Electrolyte leakage. **Table S26.** Fisher Test Raw Data for POD. **Table S27.** Fisher Test Raw Data for SOD. **Table S28.** Fisher Test Raw Data for CAT.

## Data Availability

All data generated or analyzed during this study are included in this published article and supplymentry information file.
