# Supplementary material for: Evaluation of potassium-enriched biochar and GA3 effectiveness for Improving wheat growth under drought stress
Source: BMC Plant Biol. 2023 Dec 5;23:615. doi: 10.1186/s12870-023-04613-0 (PMC10696804; doi:10.1186/s12870-023-04613-0)
Supplement: Supplementary file 1 — Additional file 1: Table S1. Overall ANOVA for Gemination (%). Table S2. Overall ANOVA for Shoot length (cm). Table S3. Overall ANOVA for Root length (cm). Table S4. Overall ANOVA for Shoot fresh weight (g). Table S5. Overall ANOVA for Shoot dry weight (g). Table S6. Overall ANOVA for Root fresh weight (g). Table S7. Overall ANOVA for Root dry weight (g). Table S8. Overall ANOVA for Chlorophyll a (mg/g). Table S9. Overall ANOVA for Chlorophyll b (mg/g). Table S10. Overall ANOVA for Total chlorophyll (mg/g). Table S11. Overall ANOVA for Electrolyte leakage (%).Table S12. Overall ANOVA for POD (units/mg protein). Table S13. Overall ANOVA for SOD (units/mg protein). Table S14. Overall ANOVA for CAT (units/mg protein). Table S15. Fisher Test Raw Data for Germination. Table S16. Fisher Test Raw Data for Shoot length. Table S17. Fisher Test Raw Data for Root length. Table S18. Fisher Test Raw Data for Shoot fresh weight. Table S19. Fisher Test Raw Data for Shoot dry weight. Table S20. Fisher Test Raw Data for Root fresh weight. Table S21. Fisher Test Raw Data for Root dry weight. Table S22. Fisher Test Raw Data for Chlorophyll a. Table S23. Fisher Test Raw Data for Chlorophyll b. Table S24. Fisher Test Raw Data for Total chlorophyll. Table S25. Fisher Test Raw Data for Electrolyte leakage. Table S26. Fisher Test Raw Data for POD. Table S27. Fisher Test Raw Data for SOD. Table S28. Fisher Test Raw Data for CAT. [file 12870_2023_4613_MOESM1_ESM.docx]

**Supplymentery Information File**

**Table S1.** Overall ANOVA for Gemination (%)

| Attributes | df | Sum of Squares | Mean Square | F Value | P Value |
| --- | --- | --- | --- | --- | --- |
| Treatments | 3 | 232.5 | 77.5 | 132.85714 | <0.0001 |
| Stress | 1 | 780.125 | 780.125 | 1337.35714 | <0.0001 |
| Interaction | 3 | 1.375 | 0.45833 | 0.78571 | 0.51365 |
| Model | 7 | 1014 | 144.85714 | 248.32653 | <0.0001 |
| Error | 24 | 14 | 0.58333 |  |  |
| Corrected Total | 31 | 1028 |  |  |  |

**Table S2.** Overall ANOVA for Shoot length (cm)

| Attributes | df | Sum of Squares | Mean Square | F Value | P Value |
| --- | --- | --- | --- | --- | --- |
| Treatments | 3 | 43.74871 | 14.5829 | 82.56177 | <0.0001 |
| Stress | 1 | 117.61945 | 117.61945 | 665.90791 | <0.0001 |
| Interaction | 3 | 2.00671 | 0.6689 | 3.78703 | 0.02348 |
| Model | 7 | 163.37487 | 23.33927 | 132.13633 | <0.0001 |
| Error | 24 | 4.23913 | 0.17663 |  |  |
| Corrected Total | 31 | 167.614 |  |  |  |

**Table S3.** Overall ANOVA for Root length (cm)

| Attributes | df | Sum of Squares | Mean Square | F Value | P Value |
| --- | --- | --- | --- | --- | --- |
| Treatments | 3 | 3.13863 | 1.04621 | 26.97652 | <0.0001 |
| Stress | 1 | 33.15015 | 33.15015 | 854.77551 | <0.0001 |
| Interaction | 3 | 0.47751 | 0.15917 | 4.10419 | 0.01745 |
| Model | 7 | 36.7663 | 5.25233 | 135.43109 | <0.0001 |
| Error | 24 | 0.93078 | 0.03878 |  |  |
| Corrected Total | 31 | 37.69707 |  |  |  |

**Table S4.** Overall ANOVA for Shoot fresh weight (g)

| Attributes | df | Sum of Squares | Mean Square | F Value | P Value |
| --- | --- | --- | --- | --- | --- |
| Treatments | 3 | 1.11454 | 0.37151 | 76.20769 | <0.0001 |
| Stress | 1 | 5.13601 | 5.13601 | 1053.54103 | <0.0001 |
| Interaction | 3 | 0.01344 | 0.00448 | 0.9188 | 0.44668 |
| Model | 7 | 6.26399 | 0.89486 | 183.56007 | <0.0001 |
| Error | 24 | 0.117 | 0.00488 |  |  |
| Corrected Total | 31 | 6.38099 |  |  |  |

**Table S5.** Overall ANOVA for Shoot dry weight (g)

| Attributes | df | Sum of Squares | Mean Square | F Value | P Value |
| --- | --- | --- | --- | --- | --- |
| Treatments | 3 | 0.13893 | 0.04631 | 58.34121 | <0.0001 |
| Stress | 1 | 0.29645 | 0.29645 | 373.48031 | <0.0001 |
| Interaction | 3 | 0.02013 | 0.00671 | 8.45144 | 5.22762E-4 |
| Model | 7 | 0.4555 | 0.06507 | 81.97975 | <0.0001 |
| Error | 24 | 0.01905 | 7.9375E-4 |  |  |
| Corrected Total | 31 | 0.47455 |  |  |  |

**Table S6.** Overall ANOVA for Root fresh weight (g)

| Attributes | df | Sum of Squares | Mean Square | F Value | P Value |
| --- | --- | --- | --- | --- | --- |
| Treatments | 3 | 0.05453 | 0.01818 | 79.30909 | <0.0001 |
| Stress | 1 | 0.17701 | 0.17701 | 772.41818 | <0.0001 |
| Interaction | 3 | 0.00156 | 5.20833E-4 | 2.27273 | 0.10585 |
| Model | 7 | 0.2331 | 0.0333 | 145.30909 | <0.0001 |
| Error | 24 | 0.0055 | 2.29167E-4 |  |  |
| Corrected Total | 31 | 0.2386 |  |  |  |

**Table S7.** Overall ANOVA for Root dry weight (g)

| Attributes | df | Sum of Squares | Mean Square | F Value | P Value |
| --- | --- | --- | --- | --- | --- |
| Treatments | 3 | 0.00566 | 0.00189 | 72.44 | <0.0001 |
| Stress | 1 | 0.04278 | 0.04278 | 1642.68 | <0.0001 |
| Interaction | 3 | 7.09375E-4 | 2.36458E-4 | 9.08 | 3.38327E-4 |
| Model | 7 | 0.04915 | 0.00702 | 269.60571 | <0.0001 |
| Error | 24 | 6.25E-4 | 2.60417E-5 |  |  |
| Corrected Total | 31 | 0.04977 |  |  |  |

**Table S8.** Overall ANOVA for Chlorophyll a (mg/g)

| Attributes | df | Sum of Squares | Mean Square | F Value | P Value |
| --- | --- | --- | --- | --- | --- |
| Treatments | 3 | 0.30618 | 0.10206 | 55.82849 | <0.0001 |
| Stress | 1 | 0.723 | 0.723 | 395.48889 | <0.0001 |
| Interaction | 3 | 0.05291 | 0.01764 | 9.64729 | 2.31379E-4 |
| Model | 7 | 1.0821 | 0.15459 | 84.55946 | <0.0001 |
| Error | 24 | 0.04388 | 0.00183 |  |  |
| Corrected Total | 31 | 1.12597 |  |  |  |

**Table S9.** Overall ANOVA for Chlorophyll b (mg/g)

| Attributes | df | Sum of Squares | Mean Square | F Value | P Value |
| --- | --- | --- | --- | --- | --- |
| Treatments | 3 | 0.17053 | 0.05684 | 119.41138 | <0.0001 |
| Stress | 1 | 0.73508 | 0.73508 | 1544.14661 | <0.0001 |
| Interaction | 3 | 0.02598 | 0.00866 | 18.19475 | <0.0001 |
| Model | 7 | 0.9316 | 0.13309 | 279.56643 | <0.0001 |
| Error | 24 | 0.01143 | 4.76042E-4 |  |  |
| Corrected Total | 31 | 0.94302 |  |  |  |

**Table S10.** Overall ANOVA for Total chlorophyll (mg/g)

| Attributes | df | Sum of Squares | Mean Square | F Value | P Value |
| --- | --- | --- | --- | --- | --- |
| Treatments | 3 | 0.93026 | 0.31009 | 99.96105 | <0.0001 |
| Stress | 1 | 2.91611 | 2.91611 | 940.0497 | <0.0001 |
| Interaction | 3 | 0.00996 | 0.00332 | 1.07052 | 0.38018 |
| Model | 7 | 3.85634 | 0.55091 | 177.59206 | <0.0001 |
| Error | 24 | 0.07445 | 0.0031 |  |  |
| Corrected Total | 31 | 3.93079 |  |  |  |

**Table S11.** Overall ANOVA for Electrolyte leakage (%)

| Attributes | df | Sum of Squares | Mean Square | F Value | P Value |
| --- | --- | --- | --- | --- | --- |
| Treatments | 3 | 144.00514 | 48.00171 | 81.54138 | <0.0001 |
| Stress | 1 | 694.71281 | 694.71281 | 1180.12128 | <0.0001 |
| Interaction | 3 | 0.44164 | 0.14721 | 0.25007 | 0.86048 |
| Model | 7 | 839.15959 | 119.87994 | 203.64223 | <0.0001 |
| Error | 24 | 14.1283 | 0.58868 |  |  |
| Corrected Total | 31 | 853.28789 |  |  |  |

**Table S12.** Overall ANOVA for POD (units/mg protein)

| Attributes | df | Sum of Squares | Mean Square | F Value | P Value |
| --- | --- | --- | --- | --- | --- |
| Treatments | 3 | 2.22616 | 0.74205 | 57.71458 | <0.0001 |
| Stress | 1 | 8.62163 | 8.62163 | 670.56332 | <0.0001 |
| Interaction | 3 | 0.39371 | 0.13124 | 10.20716 | 1.60814E-4 |
| Model | 7 | 11.2415 | 1.60593 | 124.90408 | <0.0001 |
| Error | 24 | 0.30857 | 0.01286 |  |  |
| Corrected Total | 31 | 11.55007 |  |  |  |

**Table S13.** Overall ANOVA for SOD (units/mg protein)

| Attributes | df | Sum of Squares | Mean Square | F Value | P Value |
| --- | --- | --- | --- | --- | --- |
| Treatments | 3 | 586.81916 | 195.60639 | 35.89384 | <0.0001 |
| Stress | 1 | 3235.90013 | 3235.90013 | 593.78888 | <0.0001 |
| Interaction | 3 | 265.75678 | 88.58559 | 16.25549 | <0.0001 |
| Model | 7 | 4088.47607 | 584.06801 | 107.1767 | <0.0001 |
| Error | 24 | 130.78993 | 5.44958 |  |  |
| Corrected Total | 31 | 4219.266 |  |  |  |

**Table S14.** Overall ANOVA for CAT (units/mg protein)

| Attributes | df | Sum of Squares | Mean Square | F Value | P Value |
| --- | --- | --- | --- | --- | --- |
| Treatments | 3 | 0.42266 | 0.14089 | 86.75497 | <0.0001 |
| Stress | 1 | 1.276 | 1.276 | 785.73637 | <0.0001 |
| Interaction | 3 | 0.02223 | 0.00741 | 4.56382 | 0.01148 |
| Model | 7 | 1.7209 | 0.24584 | 151.38468 | <0.0001 |
| Error | 24 | 0.03897 | 0.00162 |  |  |
| Corrected Total | 31 | 1.75987 |  |  |  |

**Table S15. Fisher Test Raw Data for Germination**

| Stress | Treatments | Mean | SD | SE of Mean | Label |
| --- | --- | --- | --- | --- | --- |
| OS | Control | 71.5 | 0.57735 | 0.28868 | h |
| OS | GA3+KBC | 78.25 | 0.95743 | 0.47871 | e |
| OS | GA3 | 75.25 | 0.95743 | 0.47871 | f |
| OS | KBC | 73.25 | 0.5 | 0.25 | g |
| NoOS | Control | 80.75 | 0.5 | 0.25 | d |
| NoOS | GA3+KBC | 88.5 | 0.57735 | 0.28868 | a |
| NoOS | GA3 | 85.5 | 1 | 0.5 | b |

**Table S16. Fisher Test Raw Data for Shoot length**

| Stress | Treatments | Mean | SD | SE of Mean | Label |
| --- | --- | --- | --- | --- | --- |
| OS | Control | 8.4725 | 0.12066 | 0.06033 | h |
| OS | GA3+KBC | 10.955 | 0.31554 | 0.15777 | e |
| OS | GA3 | 10.105 | 0.36042 | 0.18021 | f |
| OS | KBC | 9.2575 | 0.25448 | 0.12724 | g |
| NoOS | Control | 11.6025 | 0.20073 | 0.10036 | d |
| NoOS | GA3+KBC | 15.285 | 0.41964 | 0.20982 | a |
| NoOS | GA3 | 14.39 | 0.1635 | 0.08175 | b |

**Table S17. Fisher Test Raw Data for Root length**

| Stress | Treatments | Mean | SD | SE of Mean | Label |
| --- | --- | --- | --- | --- | --- |
| OS | Control | 5.16 | 0.02944 | 0.01472 | f |
| OS | GA3+KBC | 6.2875 | 0.4655 | 0.23275 | d |
| OS | GA3 | 5.6025 | 0.16741 | 0.0837 | e |
| OS | KBC | 5.3625 | 0.12738 | 0.06369 | ef |
| NoOS | Control | 7.32 | 0.19131 | 0.09566 | c |
| NoOS | GA3+KBC | 7.9075 | 0.015 | 0.0075 | a |
| NoOS | GA3 | 7.72 | 0.10677 | 0.05339 | ab |

**Table S18. Fisher Test Raw Data for Shoot fresh weight**

| Stress | Treatments | Mean | SD | SE of Mean | Label |
| --- | --- | --- | --- | --- | --- |
| OS | Control | 3.6125 | 0.03775 | 0.01887 | g |
| OS | GA3+KBC | 4.1025 | 0.08302 | 0.04151 | e |
| OS | GA3 | 3.9125 | 0.02872 | 0.01436 | f |
| OS | KBC | 3.8325 | 0.06397 | 0.03198 | f |
| NoOS | Control | 4.375 | 0.1179 | 0.05895 | d |
| NoOS | GA3+KBC | 4.905 | 0.04123 | 0.02062 | a |
| NoOS | GA3 | 4.78 | 0.08718 | 0.04359 | b |

**Table S19. Fisher Test Raw Data for Shoot dry weight**

| Stress | Treatments | Mean | SD | SE of Mean | Label |
| --- | --- | --- | --- | --- | --- |
| OS | Control | 0.34 | 0.0216 | 0.0108 | f |
| OS | GA3+KBC | 0.5725 | 0.01258 | 0.00629 | d |
| OS | GA3 | 0.5325 | 0.02986 | 0.01493 | d |
| OS | KBC | 0.445 | 0.06351 | 0.03175 | e |
| NoOS | Control | 0.615 | 0.01 | 0.005 | c |
| NoOS | GA3+KBC | 0.73 | 0.01633 | 0.00816 | a |
| NoOS | GA3 | 0.68 | 0.01826 | 0.00913 | b |

**Table S20. Fisher Test Raw Data for Root fresh weight**

| Stress | Treatments | Mean | SD | SE of Mean | Label |
| --- | --- | --- | --- | --- | --- |
| OS | Control | 0.3825 | 0.00957 | 0.00479 | h |
| OS | GA3+KBC | 0.5075 | 0.015 | 0.0075 | e |
| OS | GA3 | 0.4625 | 0.02363 | 0.01181 | f |
| OS | KBC | 0.41 | 0.02449 | 0.01225 | g |
| NoOS | Control | 0.545 | 0.01291 | 0.00645 | d |
| NoOS | GA3+KBC | 0.635 | 0.01 | 0.005 | a |
| NoOS | GA3 | 0.6075 | 0.00957 | 0.00479 | b |

**Table S21. Fisher Test Raw Data for Root dry weight**

| Stress | Treatments | Mean | SD | SE of Mean | Label |
| --- | --- | --- | --- | --- | --- |
| OS | Control | 0.165 | 0.00577 | 0.00289 | g |
| OS | GA3+KBC | 0.2125 | 0.005 | 0.0025 | d |
| OS | GA3 | 0.1925 | 0.00957 | 0.00479 | e |
| OS | KBC | 0.18 | 0 | 0 | f |
| NoOS | Control | 0.2475 | 0.005 | 0.0025 | c |
| NoOS | GA3+KBC | 0.27 | 0 | 0 | a |
| NoOS | GA3 | 0.27 | 0 | 0 | a |

**Table S22. Fisher Test Raw Data for Chlorophyll a**

| Stress | Treatments | Mean | SD | SE of Mean | Label |
| --- | --- | --- | --- | --- | --- |
| OS | Control | 1.0625 | 0.02754 | 0.01377 | f |
| OS | GA3+KBC | 1.4275 | 0.0263 | 0.01315 | c |
| OS | GA3 | 1.35 | 0.06055 | 0.03028 | d |
| OS | KBC | 1.2225 | 0.09069 | 0.04535 | e |
| NoOS | Control | 1.485 | 0.00577 | 0.00289 | c |
| NoOS | GA3+KBC | 1.6425 | 0.0263 | 0.01315 | a |
| NoOS | GA3 | 1.59 | 0.01414 | 0.00707 | ab |

**Table S23. Fisher Test Raw Data for Chlorophyll b**

| Stress | Treatments | Mean | SD | SE of Mean | Label |
| --- | --- | --- | --- | --- | --- |
| OS | Control | 0.68 | 0.00816 | 0.00408 | g |
| OS | GA3+KBC | 0.8225 | 0.02217 | 0.01109 | e |
| OS | GA3 | 0.73 | 0.00816 | 0.00408 | f |
| OS | KBC | 0.715 | 0.01 | 0.005 | f |
| NoOS | Control | 0.895 | 0.03786 | 0.01893 | d |
| NoOS | GA3+KBC | 1.1525 | 0.015 | 0.0075 | a |
| NoOS | GA3 | 1.1 | 0.02449 | 0.01225 | b |

**Table S24. Fisher Test Raw Data for Total chlorophyll**

| Stress | Treatments | Mean | SD | SE of Mean | Label |
| --- | --- | --- | --- | --- | --- |
| OS | Control | 1.7425 | 0.035 | 0.0175 | h |
| OS | GA3+KBC | 2.25 | 0.0469 | 0.02345 | e |
| OS | GA3 | 2.08 | 0.06782 | 0.03391 | f |
| OS | KBC | 1.9375 | 0.09912 | 0.04956 | g |
| NoOS | Control | 2.38 | 0.04243 | 0.02121 | d |
| NoOS | GA3+KBC | 2.795 | 0.04123 | 0.02062 | a |
| NoOS | GA3 | 2.69 | 0.03742 | 0.01871 | b |

**Table S25. Fisher Test Raw Data for Electrolyte leakage**

| Stress | Treatments | Mean | SD | SE of Mean | Label |
| --- | --- | --- | --- | --- | --- |
| OS | Control | 50.2575 | 0.2424 | 0.1212 | a |
| OS | GA3+KBC | 44.72 | 0.82728 | 0.41364 | d |
| OS | GA3 | 46.3675 | 0.27256 | 0.13628 | c |
| OS | KBC | 48.74 | 0.94099 | 0.4705 | b |
| NoOS | Control | 41.13 | 1.53677 | 0.76839 | e |
| NoOS | GA3+KBC | 35.4175 | 0.14728 | 0.07364 | h |
| NoOS | GA3 | 37.23 | 0.7422 | 0.3711 | g |

**Table S26. Fisher Test Raw Data for POD**

| Stress | Treatments | Mean | SD | SE of Mean | Label |
| --- | --- | --- | --- | --- | --- |
| OS | Control | 3.1525 | 0.07805 | 0.03902 | a |
| OS | GA3+KBC | 2.72 | 0.08287 | 0.04143 | c |
| OS | GA3 | 2.9425 | 0.02872 | 0.01436 | b |
| OS | KBC | 3.0175 | 0.04992 | 0.02496 | ab |
| NoOS | Control | 2.455 | 0.1933 | 0.09665 | d |
| NoOS | GA3+KBC | 1.4275 | 0.1652 | 0.0826 | g |
| NoOS | GA3 | 1.7825 | 0.12971 | 0.06486 | f |

**Table S27. Fisher Test Raw Data for SOD**

| Stress | Treatments | Mean | SD | SE of Mean | Label |
| --- | --- | --- | --- | --- | --- |
| OS | Control | 51.2625 | 0.81651 | 0.40826 | a |
| OS | GA3+KBC | 47.385 | 0.6143 | 0.30715 | b |
| OS | GA3 | 48.7 | 0.19026 | 0.09513 | ab |
| OS | KBC | 49.575 | 0.62846 | 0.31423 | ab |
| NoOS | Control | 40.0225 | 5.81319 | 2.90659 | c |
| NoOS | GA3+KBC | 20.625 | 0.5005 | 0.25025 | f |
| NoOS | GA3 | 25.54 | 2.60823 | 1.30412 | e |

**Table S28. Fisher Test Raw Data for CAT**

| Stress | Treatments | Mean | SD | SE of Mean | Label |
| --- | --- | --- | --- | --- | --- |
| OS | Control | 1.5025 | 0.01708 | 0.00854 | a |
| OS | GA3+KBC | 1.2675 | 0.03775 | 0.01887 | c |
| OS | GA3 | 1.3725 | 0.04349 | 0.02175 | b |
| OS | KBC | 1.46 | 0.02309 | 0.01155 | a |
| NoOS | Control | 1.1825 | 0.02062 | 0.01031 | d |
| NoOS | GA3+KBC | 0.8 | 0.07528 | 0.03764 | g |
| NoOS | GA3 | 0.96 | 0.03559 | 0.0178 | f |
